# RNA modifications in cancer and their detection: a review

**DOI:** 10.1093/jjco/hyag018

**Published:** 2026-01-30

**Authors:** Bo-Yi Yu, Hiroki Ueda

**Affiliations:** Advanced Data Science Division, Research Center for Advanced Science and Technologies, The University of Tokyo, 153-8904, 4-6-1 Komaba Meguro-ku Tokyo, Japan; Advanced Data Science Division, Research Center for Advanced Science and Technologies, The University of Tokyo, 153-8904, 4-6-1 Komaba Meguro-ku Tokyo, Japan; Health and Disease Omics Center, Chiba University, 260-8670 1-8-1 Inohana, Chuo-ku

**Keywords:** RNA modifications, non-coding RNA, nanopore direct RNA sequencing

## Abstract

Ribonucleic acid (RNA) modifications, once viewed as static structural features, are now recognized as dynamic regulators of the ‘epitranscriptome’ that shape RNA fate. In cancer, dysregulation of RNA-modification writers, erasers, and readers reprograms RNA metabolism and translation, promoting tumorigenesis, metastasis, therapy resistance, and immune evasion. Across messenger RNAs, ribosomal RNA (rRNAs), transfer (tRNAs), and diverse non-coding RNAs, aberrant modification patterns drive alternative splicing, generate onco-ribosomes, enforce codon-biased translation, and remodel gene-expression networks in a context-dependent manner. This review summarizes how major RNA modifications—including m^6^A, m^5^C, pseudouridine, inosine, and ac^4^C—and their regulators contribute to cancer biology, together with disease-associated changes in rRNA, tRNA, and regulatory non-coding RNAs. We then discuss emerging diagnostic and prognostic biomarkers, druggable nodes within the epitranscriptomic machinery, and combination strategies that integrate RNA-modification targeting with existing therapies and immunotherapy. Finally, we outline key technologies for mapping RNA modifications, comparing mass spectrometry and NGS-based chemical or antibody-enrichment approaches with the expanding capabilities of nanopore direct RNA sequencing. Recent advances in nanopore direct RNA sequencing technologies, leveraging new chemistry (e.g. RNA004) and deep-learning basecallers (e.g. Dorado), increasingly enable single-molecule, multi-modification profiling, accelerating discovery despite inherent technical challenges. Collectively, biological, clinical, and technological progress is transforming the epitranscriptome into a tractable dimension of cancer biology and a promising source of future biomarkers and RNA-targeted precision therapies.

## Teaser

**Mini abstract** This review highlights cancer-associated ribonucleic acid (RNA) modifications and their writers, erasers and readers, spanning messenger RNAs, ribosomal RNAs, transfer RNAs and non-coding RNAs, and surveys diagnostic biomarkers, therapeutic vulnerabilities, and sequencing-based detection, emphasizing nanopore direct RNA sequencing.

## Introduction

Although the discovery of pseudouridine (Ψ) in 1957 established the existence of ribonucleic acid (RNA) modifications [1], their functional significance remained elusive due to technological limitations. Recent advances—particularly in high-throughput sequencing, mass spectrometry, and chemical biology—have revealed that RNA modifications are involved in a wide range of cellular processes, including transcription, RNA stability, translation, splicing, nuclear export, and phase separation [2–5]. In cancer, these regulatory layers become widely disrupted across messenger RNA (mRNAs), ribosomal RNA (rRNAs), transfer (tRNAs), and diverse non-coding RNAs, rewiring RNA processing and translational programs. Accumulating evidence links such aberrant modification landscapes to hallmark oncogenic processes—including uncontrolled proliferation, metastatic progression, and therapeutic resistance—underscoring their emerging promise as biomarkers and actionable targets in precision oncology [6].

Crucially, while precision oncology has largely focused on somatic mutations and transcriptional dysregulation, accumulating evidence points to a pivotal layer of post-transcriptional regulation—the epitranscriptome—as playing a significant role across various aspects of cancer biology, including its initiation and progression. In tumors, aberrant RNA modifications occur across multiple RNA classes—including mRNAs, rRNAs, tRNAs, and diverse non-coding RNAs—and these alterations profoundly disrupt RNA metabolism and cellular function. Such dysregulated RNA-modification landscapes are increasingly implicated in key oncogenic processes—including tumor initiation, metastatic dissemination, and therapeutic resistance—underscoring their promise as biomarkers and actionable targets in precision oncology.

In this review, we summarize the current knowledge on how RNA modifications contribute to cancer biology, and then describe the methodologies that have enabled these discoveries. Specifically, we highlight experimental and computational strategies for detecting and quantifying RNA modifications, with particular attention to recent progress in nanopore-based direct RNA sequencing technologies.

## Ribonucleic acid modifications in cancer biology

**Figure 1:**
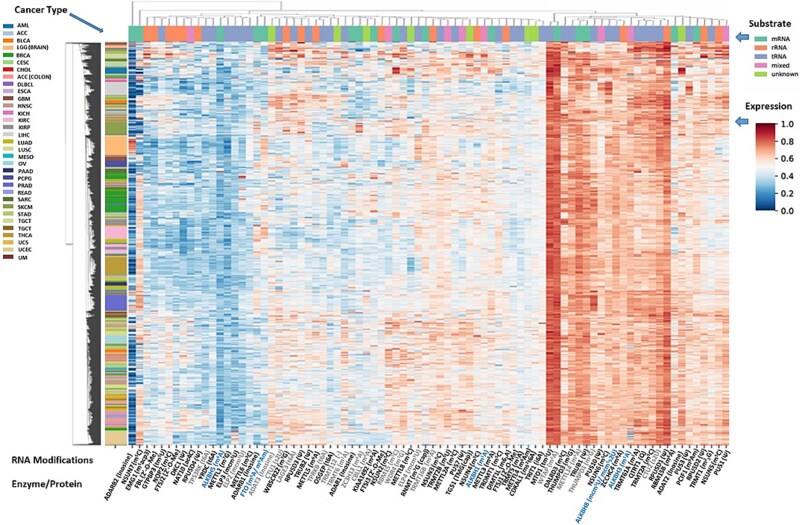
Expression landscape of RNA-modification regulators across TCGA Pan-Cancer cohorts. Hierarchical clustering of RNA-modification enzymes was performed using bulk RNA-seq data from TCGA Pan-Cancer samples. Each column represents an RNA-modification regulator, annotated by its predominant RNA substrate (mRNA, rRNA, tRNA, mixed, or unknown; color bar at the bottom). Each row corresponds to a tumor sample, with the side color bar indicating cancer type. Heatmap values show scaled expression levels (z-scores), with red and blue denoting relatively high and low expression, respectively. The clustering reveals that, even when restricted to RNA-modification regulators, tumor samples segregate into groups that broadly recapitulate their cancer types. Cancer type labels follow the standard TCGA abbreviations. RNA-modification enzymes possessing direct catalytic activity are displayed in black, whereas non-catalytic regulators (e.g., subunits) are shown in grey; eraser enzymes are indicated in blue font.

Aberrant expression of RNA modification regulatory genes—writers, erasers, and readers—is a direct and widely reported indicator of a perturbed epitranscriptomic landscape in tumors. This recurrent feature reshapes modification patterns and drives tumorigenic pathways [7–9] (also Fig. 1). While tRNAs and rRNAs harbor a wide variety of modifications whose alterations can exert broad effects on the translation of many genes, modification abnormalities detected in mRNAs tend to show a certain degree of gene specificity. In the following sections, we review major types of cancer-associated RNA modifications in mRNAs, and then summarize current knowledge on aberrant modifications in rRNAs, tRNAs, and other non-coding RNAs. Many non-coding RNAs—including miRNAs, and snoRNAs—are genomically coupled to protein-coding genes by shared promoters or intronic localization, and therefore also undergo widespread tumor-associated expression changes alongside mRNAs. While comprehensive studies of RNA modifications in the field of oncology have remained limited, large-scale cancer genomic cohorts such as TCGA and ICGC have reported aberrant expressions of various RNA modification-related genes (RMRGs). Fig. 1 shows clustering of RNA modification–related enzymes, including writers, and erasers, using TCGA bulk RNA-seq data, and highlights multiple expression patterns that are consistent with previously reported cancer-associated dysregulation of RNA modification machinery across diverse tumor types. Distinct tumor groups may be suggested based on characteristic expression patterns of RNA-modification regulators, for example, acute myeloid leukemia (AML) appears to show higher expression of RBM15 (m^6^A writer complex). Liver hepatocellular carcinoma (LIHC) may exhibit increased levels of PUS3 and TRUB2 (pseudouridine synthases), as well as NSUN6 (m^5^C writer), whereas NSUN7 (m5c-related) tends to show lower expression. Thyroid carcinoma (THCA) may be associated with reduced METTL8 (m^3^C writer) expression. Skin cutaneous melanoma (SKCM) may also show decreased NSUN7 (m^5^C-related) expression. These observations are associated with previous reports demonstrating that dysregulated RNA modification regulators contribute to oncogenic progression across multiple cancer contexts. For example, key regulators such as IGF2BP3, CFI, and ELF3 exhibit strong, cancer type–specific prognostic value. In particular, in glioblastoma, tumors with higher expression of RNA modification–related regulators are characterized by markedly increased infiltration of M1 macrophages, together with widespread upregulation of immune checkpoint molecules, including TNFRSF18, LAG3, and PVR, indicating a profoundly remodeled and immunosuppressive tumor microenvironment associated with poorer clinical outcome [10]. Similarly, gastric cancer studies have revealed distinct RMRG subtypes associated with tumor microenvironment remodeling, immune infiltration, and prognosis, with MAMDC2 identified as a functional modulator of tumor cell proliferation and migration [11]. Collectively, these findings reinforce that cancer-associated dysregulation of RNA modification machinery is not only widespread but also functionally relevant to tumor progression, immune microenvironment remodeling, and patient prognosis, supporting the emerging translational potential of RNA epitranscriptomic regulation in oncology. However, analyses based solely on the gene expression profiles of RNA modification enzymes and reader proteins remain insufficient to fully elucidate the complexity of these phenomena. Further technical advances in comprehensive RNA modification mapping, improvements in analytical resolution, and large-scale cohort-based studies will be essential for uncovering the biological and clinical significance of RNA epitranscriptomic regulation in cancer.

As summarized in Table 1, many mRNA modifications, most notably m6A, are regulated through a dynamic ‘writer-eraser-reader’ system. However, it should be noted that for many other modifications, particularly those in non-coding RNAs like tRNA and rRNA, this framework may not fully apply, as they often lack known erasers or function primarily by altering RNA structure rather than recruiting specific readers. Because some modifications possess not only writers but also erasers, the modification status of RNA is considered to be dynamically regulated. However, to date, bona fide erasers have been identified for only a limited number of modifications—most prominently m^6^A, and to some extent m^1^A and m^5^C. In contrast, the vast majority of RNA modifications are intrinsically irreversible and function without active removal mechanisms throughout the lifespan of the RNA molecule.

**Table 1:** Writer, eraser, reader of major RNA modification.

Modification	Writer(s)	Eraser(s)	Reader(s)	Notes
m^6^A	METTL3^*^, METTL14, WTAP, METTL16^*^ VIRMA (KIAA1429), RBM15/15B, ZC3H13	FTO ALKBH5	YTHDF1/2/3, YTHDC1/2 IGF2BP1/2/3, HNRNPC HNRNPA2B1, FMR1	Best-characterized reversible modification Regulates transcription, splicing, export, translation, and decay
Ψ	PUS1–PUS10^*^,, TRUB1/2^*^, RPUSD 1–4^*^, DKC1^*^ + H/ACA box snoRNAs	–	*–*	Added by stand-alone PUS enzymes and H/ACA snoRNPs Abundant in rRNA, tRNA, snRNA
Inosine	ADAR1, ADAR2	–	*–*	A → I changes base-pairing (I read as G) Recodes amino acids
Nm	FBL ^*^ (C/D box snoRNP) FTSJ1/2^*^, CMTR1/2^*^ MTr1–3^*^, BCDIN3D^*^	–	*–*	snoRNA-guided modification stabilizes rRNA/snRNA and improves fidelity
m^5^C	NSUN1–7^*^, DNMT2^*^	TET2	ALYREF, YBX1	Regulates mRNA stability, export, and translation
m^7^G	METTL1^*^–WDR4 (tRNA) RNMT^*^–RAM (mRNA cap)	–	eIF4E, eIF4E2	Abundant internally in tRNA and at the mRNA cap Recently found in internal mRNA
m^1^A	TRMT6/TRMT61A^*^ TRMT61B^*^, TRMT10C^*^	ALKBH1 ALKBH3	YTHDF1/2/3, YTHDC1 (putative)	Occurs in tRNA, rRNA, and mRNA Partially reversible
m^6^Am	PCIF1^*^ (CAPAM)	FTO	YTH proteins may bind weakly	Found at the transcription-start-associated cap structure (m7G–m6Am) Regulates mRNA stability and translation initiation
ac^4^C	NAT10^*^	–	–	Regulates mRNA stability and translation

‘^–^’ indicates no regulator identified to date; under the ‘Writers’ column, asterisks (^*^) denote the subunits possessing catalytic activity within the respective complexes, whereas others function as non-catalytic cofactors essential for complex stability or substrate recruitment. The ‘writer-eraser-reader’ paradigm is most prominently established for dynamic mRNA modifications such as m6A. For many constitutive modifications in tRNA, rRNA, and snRNA, erasers or specific readers may not exist or have not yet been identified.

In this table, we include only representative mRNA-modifying enzymes. By contrast, tRNAs [12] and rRNAs [13] harbor more than 150 distinct chemical modifications, each typically installed by dedicated writer enzymes, and in some cases—such as m^1^A [14, 15] or the reversible Up47 modification in thermophilic archaea [16]—subject to enzymatic removal. However, because the majority of these modifications are thought to contribute to RNA structural integrity and translational accuracy, whether they undergo dynamic regulation remains to be resolved, and most currently have no identified erasers or readers. Fig. 1 shows a clustering analysis of modification-related genes using TCGA Pan-Cancer data, demonstrating that cancer types can be broadly classified even when using only the expression profiles of RNA-modification regulators.

Despite the rapidly expanding body of literature linking RNA modifications to cancer, a major limitation of the current literature is that many cancer-associated RNA-modification changes are supported primarily by correlative evidence (expression/prognosis associations and peak-level maps), whereas site-resolved causal links—i.e. demonstrating that modification at a defined nucleotide directly drives a specific oncogenic phenotype—remain comparatively scarce. This gap reflects technical constraints in accurately quantifying stoichiometry and perturbing individual modification sites without affecting the broader RNA-processing machinery.

### Messenger Ribonucleic acid modifications

#### N^6^-methyladenosine

As one of the most abundant modifications on mRNAs (0.4%—0.7% of m^6^A/A ratio) [17], m^6^A has emerged as a key epitranscriptomic mark involved in carcinogenesis [17]. Notably, these methylated adenosines influence diverse mRNA functions such as stability, localization, splicing, and translation [18]. By shaping the fate of RNAs in cells, m^6^A has opened up new avenues for understanding the molecular mechanisms that drive the transition from normal to malignant states.

A growing number of studies have documented cases in which m^6^A regulators are significantly upregulated or downregulated, reshaping the modification landscape and contributing to tumorigenic processes (Table 2). For instance, METTL3, which is the catalytic core of the m^6^A methyltransferase complex, is widely reported as an oncogenic factor in multiple cancers. In esophageal squamous cell carcinoma (ESCC), aberrantly increased METTL3 expression promotes YTHDF-dependent *APC* mRNA degradation, enhancing β-catenin signaling and driving ESCC progression [19]. In tumor infiltrating myeloid cells, the METTL3 upregulation induced by histone lactylation augments the translational efficiency of JAK1 protein and fosters an immunosuppressive tumor microenvironment in a m^6^A-dependent manner [20]. Moreover, one study demonstrated that elevated glycolysis in colorectal cancer (CRC) cells is driven by METTL3-mediated m^6^A deposition on *HK2* and *SLC2A1* mRNAs, leading to tumorigenesis [21].

**Table 2:** Dysregulation of m^6^A mRNA modification in cancers.

Cancer type	Target	Mechanisms of carcinogenesis	Ref.
ESCC	APC	METTL3-mediated m^**6**^A promotes APC mRNA degradation and enhances β-catenin signaling	[19]
CRC, etc.	JAK1	METTL3-mediated m^**6**^A promotes JAK1 protein translation and tumor immune suppression	[20]
CRC	HK2, SLC2A1	METTL3-mediated m^**6**^A promotes stability of glycolytic transcripts	[21]
LLC, etc.	EBI3	METTL14 deficiency in tumor-associated macrophages promotes T-cell dysfunction	[22]
CRC	SOX4	METTL14-mediated m^**6**^A suppresses EMT and cancer metastasis	[23]
BC, etc.	WNT5A, etc.	FTO depletion activates Wnt signaling and promotes cancer progression	[25]
GBM	GCLM	Nuclear localization of ALKBH5 decreases m^**6**^A on GCLM mRNA and inhibits ferroptosis	[26]
CRC, etc.	CX3CL1	YTHDF2 reduces CX3CL1 mRNA stability and promotes tumor immune evasion	[27]
DLBCL	ACER2	YTHDF2 stabilizes ACER2 mRNA and regulates ceramide catabolism to promote cancer progression	[28]
AML	MYC	YTHDC1 forms nuclear condensates via liquid–liquid phase separation to protect target mRNAs from degradation	[29]

^a^ESCC: esophageal squamous cell carcinoma, CRC: colorectal cancer, LLC: Lewis lung carcinoma, BC: breast cancer, GBM: glioblastoma, DLBCL: diffuse large B-cell lymphoma, AML: acute myeloid leukemia.

METTL14, the RNA-binding subunit of the m^6^A methyltransferase complex, exhibits context-dependent functions in cancer. Dong *et al.* revealed a METTL14-dependent immunoregulatory program in tumor-associated macrophages, and loss of METTL14 in macrophages contributes to T-cell dysfunction and tumor development [22]. In CRC, Chen *et al.* identified a negative correlation between METTL14 expression and patient overall survival based on TCGA datasets [23]. Mechanistically, METTL14 installs m^6^A marks on *SOX4* mRNA to promote YTHDF2-mediated degradation, suppressing epithelial-to-mesenchymal (EMT) and cancer metastasis. Conversely, a recent study showed that a gain-of-function R298P mutation in METTL14 shifts its substrate preference toward non-canonical GGAU motifs, generating an aberrant m^6^A methylation landscape that promotes cancer cell migration and invasion [24]. Together, these results highlight the dualistic roles of METTL14, acting either as a tumor suppressor or as an oncogenic driver in a context-dependent manner.

In addition to m^6^A writers, dysregulation of m^6^A erasers and readers has been linked to carcinogenesis. Jeschke *et al.* demonstrated that FTO depletion elevates m^6^A levels on Wnt signaling related mRNAs, including *WNT5A*, *FZD1*, and *MARK2*, promoting cancer cell proliferation and invasion [25]. Treatment with the Wnt inhibitor iCRT3 alleviates the growth of FTO-depleted tumors. In glioblastoma, Lv *et al.* showed that EGFR signaling induces nuclear localization of the m^6^A demethylase ALKBH5, which reduces m^6^A modification level on *GCLM* mRNA and limits its YTHDF2-dependent decay, upregulating GCLM expression and protecting cells from ferroptosis [26]. Notably, ALKBH5 inhibitors synergize with the EGFR inhibitor erlotinib to reinforce the anti-glioblastoma activity. Moreover, the role of YTHDF2 has also been linked to tumor immune response [27]. Xiao *et al.* demonstrated that YTHDF2 binds to m^6^A sites in the 3′ UTR of *CX3CL1* mRNA and reduces mRNA stability in cancer cells, thereby suppressing the immune cell recruitment. YTHDF2 depletion diminished tumor immune evasion, and anti-PD1 therapy benefited from combination with a YTHDF2 inhibitor. In diffuse large B-cell lymphoma (DLBCL), YTHDF2 regulates ceramide catabolism by recognizing m^6^A sites on *ACER2* mRNA and increasing its stability [28]. Decreased ceramide level in DLBCL contributes to DLBCL progression, and loss of YTHDF2 enhances the response of DLBCL cells to chemotherapeutic agents such as ibrutinib and venetoclax. Notably, findings from these pharmacological and genetic inhibition studies suggest that therapeutic vulnerabilities created by aberrant m^6^A eraser and reader activities may serve as promising targets for cancer treatment.

Building on canonical roles, recent evidence further expands the functional landscape of m^6^A in cancers. Intriguingly, one study reported that m^6^A is also involved in liquid–liquid phase separation (LLPS) during leukemogenesis [29]. In AML, YTHDC1 has been demonstrated to form nuclear condensates in an m^6^A-dependent mechanism. Nuclear YTHDC1 droplets generated via LLPS protect target mRNAs such as *MYC* from degradation, supporting leukemogenesis in AML. In summary, the m^6^A machinery redefines RNA fate and remodels malignant phenotypes across diverse cancers.

#### Pseudouridine (Ψ)

Previous studies have confirmed that pseudouridine is not only present in non-coding RNAs such as tRNA and rRNA, but also abundant in mRNA [30]. However, the role of pseudouridine in regulating mRNA fate and disease progression are only beginning to be elucidated, with emerging evidence suggesting that dynamic installation of pseudouridine by pseudouridine synthase (PUS) family enzymes can influence mRNA stability, translation, and splicing [31]. Early studies reported that pseudouridine levels are increased in the serum and urine of patients with hepatocellular carcinoma (HCC) [32]. In this context, a recent study revealed that dysregulated mRNA pseudouridylation contributes to the abnormally elevated protein expression of oncogenes in HCC [32]. Analyses of The Cancer Genome Atlas (TCGA) datasets further demonstrated that PUS1 is significantly upregulated in HCC tissues and high PUS1 expression correlates with poor overall survival. Extending these observations, Xiao *et al.* adapted a fusion protein based method to identify PUS1 target transcripts in cancer cells, revealing key oncogenic mRNAs such as IRS1 and c-MYC are among its direct targets for translational modulation. By contrast, PUS7 is transcriptionally regulated by MYC/MYCN in MYC family-driven cancers, and in turn supports cancer cell growth by inducing pseudouridylation of MCTS1 as well as ATF4 mRNAs under cellular stress conditions [33]. In striking contrast to these oncogenic activities, PUS7-dependent pseudouridylation of ALKBH3 reinforces its tumor-suppressor activity in gastric cancer by enhancing its protein translation [34]. Notably, these findings illustrate that pseudouridylation exhibits context-specific regulation in cancer (Table 3), highlighting a complex regulatory mechanism that remains to be comprehensively investigated.

**Table 3:** Dysregulation of other mRNA modifications in cancers.

Cancer type	Modification	Target	Mechanisms of carcinogenesis	Ref.
HCC	Ψ	IRS1, etc.	PUS1 installs Y on cancer-related transcripts and regulates protein translation	[32]
NB	Ψ	MCTS1, ATF4	MYC family proteins regulate PUS7-dependent Y and promote cancer cell growth	[33]
GC	Ψ	ALKBH3	PUS7-dependent Y promotes ALKBH3 translation for tumor suppression	[34]
NSCLC	m^5^C	QSOX1	The NSUN2-driven m^5^C hypermethylation of QSOX1 promotes drug resistance	[37]
AML	m^5^C	FSP1	The NSUN2-mediated m^5^C modification suppresses ferroptosis	[38]
AML, CML	m^5^C	EZH2, etc.	SRSF2 binds to m^5^C-modified leukemia-associated transcripts to regulate splicing	[39]
AML	m^5^C	TSPAN13	TET2 loss leads to m^5^C hypermethylation and promotes leukemia stem cell homing	[40]
CML	I	MDM2	A-to-I editing protects MDM2 from miRNA binding	[44]
HCC, etc.	I	AZIN1	A-to-I editing enhances functional potency of AZIN1	[45]
BC	I	GABRA3	A-to-I editing attenuates GABRA3-mediated AKT activation	[46]
BC, OC	m^1^A	CSF1	ALKBH3-mediated m^1^A demethylation increases CSF1 expression	[48]
CC	m^1^A	ATP5D	ALKBH3-mediated m^1^A demethylation promotes glycolysis.	[49]
NB	m^1^A	SST	TRMT6-dependent m^1^A modification induces SST expression	[50]
GBM	Nm	KAT6B	The SNORD17–KAT6B–ZNF384 axis regulates vasculogenic mimicry	[54]
EC	Nm	PARP1	The SNORD104-PARP1 axis promotes cancer growth	[55]
PCa	Nm	PSMD13, etc.	Internal Nm sites on cancer-related transcripts regulate mRNA stability	[56]
CC, etc.	m^7^G	GSK3B, etc.	QK17 recognizes m7G and potentially regulates chemoresistance related targets.	[58]
GBM	m^7^G	TP53	IGF2BP3 binds and promotes mRNA decay of TP53	[59]
CRC	m^6^Am	FOS	PCIF1-mediated m^6^Am promotes FOS upregulation and FOS-dependent TGF-b production	[60]
NB	m^6^Am	ATF3	m6^6^m modulates neuroblastoma differentiation under all-trans-retinoic-acid treatment	[62]
AML	ac^4^C	SLC1A4, etc.	NAT10-mediated ac^4^C regulates serine uptake and biosynthesis pathways	[64]
NPC	ac^4^C	DDX5, etc.	NAT10-mediated ac^4^C suppresses T-cell immunity	[65]
BLCA	ac^4^C	AHNAK	NAT10-mediated ac^4^C promotes DNA damage repair and cisplatin resistance	[66]

^a^HCC: hepatocellular carcinoma, NB: neuroblastoma, GC: gastric cancer, BC: breast cancer, AML: acute myeloid leukemia, PCa: prostate cancer, CRC: colorectal cancer, CC: cervical cancer, OC: ovarian cancer, GBM: glioblastoma, EC: endometrial cancer, NSCLC: non-small cell lung cancer, CML: chronic myeloid leukemia, NPC: nasopharyngeal carcinoma, BLCA: bladder cancer

#### 5-methylcytosine

While DNA 5mC methylation has been well characterized, RNA m^5^C modification is still at an early stage of investigation [35]. Among m^5^C writers, the NOP2/Sun RNA methyltransferase (NSUN) proteins have been extensively explored for their catalytic roles in installing m^5^C on mRNA [36]. Recently, aberrant m^5^C patterns arising from NSUN protein dysregulation have been frequently reported. In EGFR tyrosine kinase inhibitor (EGFR-TKI) resistant non-small cell lung cancer cells, Wang *et al.* showed that NSUN2-driven m^5^C hypermethylation of *QSOX1* mRNA promotes drug resistance to the EGFR inhibitor gefitinib [37]. Knockdown of NSUN2 or QSOX1 attenuated cancer cell proliferation and induced cell apoptosis, supporting a potential role of m^5^C in targeted therapy. In addition to evidence from solid tumors, NSUN2 has been implicated in shaping cell death pathways in hematologic malignancies [38]. NSUN2-mediated m^5^C modification stabilizes FSP1, which in turn suppresses ferroptosis signaling in AML cells. Pharmacological inhibition of NSUN2 sensitizes AML cells to ferroptosis, and the combination of an NSUN2 inhibitor with conventional chemotherapies demonstrated synergistic anti-leukemic activity. Interestingly, another study indicated that NSUN2-mediated m^5^C regulates cancer cell behavior through a splicing-dependent mechanism [39]. Ma *et al.* reported that, in addition to canonical RNA m^5^C readers such as YBX1 and ALYREF, the splicing factor SRSF2 recognizes m^5^C modified mRNAs and reshapes the splicing landscape in leukemia. In leukemia patient cohorts, NSUN2 deficiency and SRSF2 mutation were linked to poor prognosis, reinforcing the clinical relevance of m^5^C modification. Dysregulation of m^5^C in cancer is not restricted to writer dysfunction, as loss of the m^5^C eraser TET methylcytosine dioxygenase 2 (TET2) can also drive leukemogenesis [40]. Li *et al.* demonstrated that TET2 deficiency contributes to aberrant upregulation of m^5^C in *TSPAN13* mRNA, stabilizing the transcript and then activating the CXCR4/CXCL12 pathway to promote the homing of leukemia stem cells. Collectively, these findings suggest that the m^5^C machinery represents a promising source of cancer biomarkers and therapeutic targets (Table 3).

#### Inosine

Adenosine-to-inosine (A-to-I) RNA editing predominantly occurs in double-stranded RNAs (dsRNAs) due to the specific binding of the catalytic enzymes adenosine deaminases acting on RNA (ADARs) [41]. A-to-I editing alters base pairing, reshapes local and long-range RNA structures, and can resolve G-quadruplexes [42, 43]. In most cases, the resulting inosines are found in non-coding regions, such as introns and 3′ untranslated region (3′ UTR) [41]. One well-characterized mechanism by which A-to-I editing contributes to tumorigenesis is the alteration of microRNA-mRNA interactions. For instance, ADAR1 has been reported to edit the 3′ UTR of *MDM2* to protect it from miR-155-mediated repression, ultimately leading to malignant progenitor propagation [44]. Beyond non-coding region events, non-synonymous recoding events arising from A-to-I editing can reshape oncogenic signaling pathways. RNA editing of AZIN1 is one of the most thoroughly characterized examples and has been reported in a wide range of malignancies. ADAR1-mediated serine-to-glycine conversion induces a conformational change and nuclear translocation of AZIN1, resulting in functional potency in driving cancer progression [45]. Furthermore, Gumireddy *et al.* showed that an A-to-I RNA editing–induced isoleucine-to-methionine substitution in GABRA3 attenuates AKT activation in breast cancer, thereby reducing breast cancer metastasis [46]. Overall, these observations position A-to-I editing as an essential regulator in cancer biology (Table 3).

#### N^1^-methyladenosine

Despite the well-characterized m^1^A sites on tRNA over the past decades, its presence on mRNA was first identified in 2016 [47]. Compared to m^6^A, m^1^A is about tenfold less abundant, accounting for ~0.015% to 0.054% of the total m^1^A/A ratio in the mRNA transcriptome [47]. Although the functional roles of m^1^A in mRNA remain largely unexplored, emerging studies indicate its importance in cancer biology (Table 3). In breast and ovarian cancers, the m^1^A eraser ALKBH3 has been demonstrated to stabilize *CSF1* mRNA in an m^1^A-dependent manner, leading to poor prognosis [48]. In addition, a recent study revealed that m^1^A modulates the glycolytic metabolism in cervical cancer models [49]. The ALKBH3-mediated m^1^A demethylation within the exonic region of *ATP5D* promotes its protein translation, facilitating glycolysis and cancer cell proliferation. Conversely, elevated m^1^A levels have also been linked to cancer progression, as reported in neuroblastoma. Upregulation of the m^1^A writer TRMT6 induces m^1^A modification of *SST* mRNA, resulting in its downregulation and malignancy [50]. Further investigations need to be conducted to clarify the context-dependent roles of m^1^A in tumor progression.

#### 2′-O-methylation

Early investigations of Nm primarily focused on its presence and functional roles in non-coding RNAs and the 5′ cap of mRNA [51]. Transcriptome-wide analysis, however, has provided evidence that Nm is also present in the coding region of mRNA [52]. While Nm on rRNA has been implicated in tumorigenesis [53], the involvement of mRNA Nm in cancer is far less well characterized. To address this gap, several studies have started to investigate how mRNA Nm may contribute to cancer progression (Table 3). In glioblastoma cells, Cui *et al.* characterized a SNORD17–KAT6B–ZNF384 regulatory axis that features an Nm site in *KAT6B* mRNA [54]. Mutation of the Nm sites led to increased KAT6B protein levels, and overexpression of KAT6B suppressed vasculogenic mimicry and glioblastoma cell proliferation. Another Nm-dependent mechanism has been described in endometrial cancer [55]. In this context, Nm on *PARP1* mRNA increases its stability and supports the oncogenic role. Applying a machine-learning model to long-read RNA sequencing data, Li *et al.* systematically mapped internal Nm sites on mRNA in prostate cancer cells [56]. Notably, they identified Nm enrichment on a cancer-related gene *PSMD13* and demonstrated that these modifications are positively related to mRNA stability. Together, these findings suggest that internal Nm modifications function as promising epitranscriptomic codes that regulate cancer cell behavior.

#### 7-methylguanosine

As a fundamental component of the 5′ cap, m^7^G prevents mRNA from degradation and facilitate translation initiation [57]. In addition to this canonical role, emerging evidence has demonstrated that METTL1-mediated m^7^G can be installed internally within mRNAs at low stoichiometry (0.02%—0.05% of m^7^G/G ratio) [58]. Zhao *et al.* recently revealed a Quaking protein-centered mechanism that links internal m^7^G to cancer cell stress responses [58]. Specifically, QK17, a member of the Quaking protein family, recognizes internal m^7^7G-modified transcripts and translocates them into stress granules. Under doxorubicin-induced stress conditions, QKI7-dependent sequestration of internal m^7^7G-modified transcripts dampens the translation of chemoresistance-related targets, including Hippo pathway components such as GSK3B and TEAD1. Consistent with this observation, overexpression of QK17 lowers chemoresistance and enhances the efficacy of chemotherapy in cancer cells. In glioblastoma, one recent study showed that IGF2BP3 binds to internal m^7^7G sites in the 3′ UTR of *TP53* mRNA and promotes its decay, leading to tumorigenesis and chemoresistance [59]. Collectively, these studies reveal a noncanonical and cancer-relevant dimension of m^7^G biology (Table 3), encouraging a broader view of how RNA modifications regulate oncogenic cellular processes.

#### N^6^–2′-O-dimethyladenosine

Positioned next to the cap structure, m^6^Am occurs in about 30% of mRNAs and serves as an essential regulatory modification [60]. Two identified regulators of m^6^Am, the writer PCIF1 and the eraser FTO, have both been reported to be dysregulated in CRC. Relier *et al.* reported that FTO deficiency results in elevated m^6^Am levels in CRC cells, contributing to stem-like characteristics and chemoresistance [61]. In parallel, Wang *et al.* focused on the role of PCIF1 and revealed that PCIF1 loss attenuates CRC malignancy by disrupting the FOS-mediated upregulation of TGF-β [60]. Interestingly, one recent study identified PCF11 as a previously unrecognized m^6^Am-specific reader and demonstrated that m^6^Am modulates neuroblastoma differentiation [62]. Given the limited mechanistic insights into m^6^Am regulation, efforts are underway to define its functional relevance in cancer progression (Table 3).

#### N^4^-acetylcytidine

Accumulating evidence indicates that ac^4^C, recently recognized as one of mRNA modifications (Table 3), plays an indispensable role in mRNA metabolism [63]. This cytidine acetylation is predominantly catalyzed by N-acetyltransferase 10 (NAT10), thereby modulating mRNA stability and translational efficiency. One recent study revealed that ac^4^C regulates key components of the serine uptake and biosynthesis pathway, such as SLC1A4, HOXA9, and MENIN, at the protein level in AML [64]. Importantly, the NAT10 inhibitor fludarabine exhibited robust anti-leukemic activity in both in vitro and in vivo AML models, underscoring its therapeutic potential. In parallel, ac^4^C has been implicated in remodeling cancer immune responses [65]. Xie *et al.* demonstrated that, in nasopharyngeal carcinoma (NPC), NAT10-mediated ac^4^C installation on DDX5 dampens T-cell-mediated immunity via HMGB1 upregulation. Building on this insight, targeting NAT10 was shown to improve tumor response to anti-PD-1 therapy. Moreover, ac^4^C is associated with chemotherapy resistance [66]. Cisplatin-induced ac^4^C modification was observed in bladder cancer, where NAT10-mediated ac^4^C deposition stabilizes *AHNAK* mRNA and promotes cisplatin resistance by stimulating DNA damage repair responses.

### Ribosomal ribonucleic acid modifications and onco-ribosomes

In cancer, aberrant rRNA modifications are increasingly recognized as drivers of ‘onco-ribosomes’ [67] that reprogram translation. Site-specific changes—most notably altered 2′-O-methylation (2′-O-Me) and pseudouridylation (Ψ), which together account for more than 95% of known rRNA modifications—can shift ribosome function and fidelity, thereby biasing the translatome toward pro-tumorigenic programs [68, 69]. Most of these modifications are catalyzed by small nucleolar ribonucleoproteins (snoRNPs): C/D box snoRNPs guide 2′-O-Me via the methyltransferase fibrillarin (FBL), whereas H/ACA box snoRNPs direct Ψ formation through dyskerin (DKC1). Cancer-associated alterations in snoRNP expression or target specificity remodel the rRNA modification landscape, contributing to the emergence of onco-ribosomes with selective translational outputs [60–62].

For example, in AML, elevated 2′-O-methylation at 18S position G1447 and U116—driven by increased FBL expression and aberrant activation of the corresponding snoRNAs SNORD127 and SNORD42A—has been shown to support leukemic stem-cell activity and proliferation [71, 72]. In solid tumors, snoRNA-mediated Ψ alterations also play prominent roles: loss of SNORA24 accelerates hepatocellular carcinoma in cooperation with oncogenic RAS [73], whereas SNORA81 upregulation in ovarian cancer increases Ψ at 28S U4606 [74]. In breast cancer, clusters of hyper-modified Ψ residues at ribosomal peripheries contribute to ribosome heterogeneity and stratify patient subgroups [75]. Collectively, these observations extend the concept that ribosome heterogeneity contributes to malignant phenotypes by reshaping translational control [68].

These rRNA modifications occur not only in regions interacting with tRNA but also throughout the ribosomal periphery, where they subtly alter rRNA folding, inter-subunit bridges, and factor-binding sites. Such structural remodeling can shift translation modes—for example, by impairing internal ribosome entry site (IRES)-dependent translation of tumor suppressors such as p53 and p27, a phenomenon observed in DKC1-deficient cells [9, 10]. Thus, altered pseudouridylation and ribose methylation can reprogram the balance between cap-dependent and IRES-mediated translation, favoring oncogenic mRNA translation under stress or hypoxia. Beyond ribose-based modifications, several base modifications of rRNA have emerged as cancer drivers. A representative example is m^1^acp^3^Ψ1248 in 18S rRNA, a composite modification in the decoding center essential for ribosome stability and fidelity; its loss—detected across >20 tumor types and in ~50% of CRCs—correlates with enhanced translation of ribosomal proteins and activation of E2F-driven proliferative programs [76]. Another example is m^6^A1832 in 18S, installed by METTL5 in cooperation with TRMT112; this mark is elevated in breast, hepatocellular, and intrahepatic cholangiocarcinomas, where it promotes selective translation of oncogenic mRNAs [77]. In addition, m^5^C modifications further diversify rRNA function: NOP2/NSUN1 methylates 28S C4447 and scaffolds C/D snoRNP assembly during ribosome biogenesis [78], whereas NSUN5 targets 28S C3782; its silencing in gliomas removes this mark, suppresses global translation, and induces stress-adaptive programs with prognostic significance [79].

Taken together, rRNA modifications generate ribosome heterogeneity and influence mRNA selectivity, thereby shaping tumor biology. These alterations provide a mechanistic basis for oncogenesis and present potential biomarkers and therapeutic targets.

### Transfer ribonucleic acid modifications

Accumulating evidence suggests that aberrant tRNA expression contributes to cancer progression [80], and that tRNA-modifying enzymes function as active drivers of tumor progression and therapy resistance rather than passive byproducts of malignant transformation [81]. Alterations in tRNA expression and modification can reshape gene-specific translational efficiencies, leading to enhanced translation of oncogenes and reduced translation of tumor-suppressor genes in cancer [82, 83]. For example, codon usage that favors oncogene translation has been shown to influence KRAS-driven tumorigenesis [84]. Methylation-based RNA modifications—including m^1^A, m^5^C and m^7^G [14]—represent major classes implicated in tumor initiation, progression and metastasis. A representative example is the METTL1/WDR4-mediated m^7^G tRNA modification, which enhances the translation of oncogenic and cell-cycle–related mRNAs—including Cyclin D1, Cyclin D2, Cyclin D3, Cyclin E1, Cyclin A2, CDK6, CDK8, and MAPK-related targets such as EGFR and VEGFA—across multiple tumor types (e.g. head and neck, lung, liver, colon, and bladder cancers, as well as teratoma). This effect occurs in a codon-dependent manner decoded by m^7^G-modified tRNAs [85, 86].

As rapidly proliferating tumor cells upregulate both transcription and translation, they depend on precise mechanisms that fine-tune decoding fidelity. tRNA modifications are central to this translational control and become especially important in cancer. For instance, U34-modifying enzymes within the Elongator–CTU–ALKBH8 pathway mediate metabolic reprogramming and therapy resistance: by promoting efficient decoding of U34-dependent codons (such as GAA, AAA, and CAA), upregulation of these enzymes enhances the translation of targets like HIF1A, boosts glycolytic flux in melanoma cells, and ultimately drives resistance to MAPK inhibitors [87], whereas ALKBH8 overexpression promotes tumor growth and angiogenesis in bladder cancer [88]. Moreover, simultaneous deletion of NSUN2—a major tRNA m^5^C writer—and METTL1 markedly sensitizes cancer cells to the chemotherapeutic agent 5-fluorouracil (5-FU) [89]. Recent CRISPR-based functional screens have further uncovered a synthetic vulnerability between U34 tRNA modification and mTORC1 inhibition [90], indicating that combined targeting of these pathways can selectively impair tumor-cell survival.

Recent work shows that mcm^5^s^2^U wobble-uridine modification is elevated in tumors and enhances the efficient translation of m^6^A-containing mRNAs, thereby boosting global protein synthesis and supporting tumor progression. At the same time, these wobble modifications prevent ribosome stalling on m^6^A-marked codons, protecting such transcripts from ribosome collision–induced no-go decay. Consequently, loss of mcm^5^s^2^U destabilizes a specific subset of mRNAs in a codon- and modification-dependent manner [91]. From this perspective, RNA-modification in tRNA holds strong potential as cancer biomarkers, therapeutic targets and classify cancer type.

### Micro ribonucleic acid modification

A-to-I RNA editing can reconfigure target sites not only in mRNAs but also within miRNAs, thereby redirecting target specificity in cancer. It can also alter miRNA maturation. Loss of ADAR1/2 leads to dysregulated miRNA processing (e.g. miR-455, miR-26a, miR-221/222) [84–86] and seed editing that reshapes targetomes (e.g. miR-200b, miR-589) [94, 95]. Editing can also generate isoforms with distinct target preferences, exemplified by miR-27a-3p, whose edited form retains MET binding but loses EGFR targeting and alters cellular phenotypes [96].

Beyond A-to-I editing, interactions between m^6^A modification and miRNAs further influence cancer progression. miRNAs can directly target RNA-modification regulators: for example, *miR-33a* suppresses proliferation of NSCLC cells by targeting METTL3 mRNA [97]. miR-145 targets YTHDF2, and miR-346 downregulates YTHDF1, thereby modulating m^6^A-dependent mRNA stability [98]. Conversely, oncomiRs such as miR-221/222 promote tumor growth by repressing targets like PTEN [99], illustrating how miRNA dysregulation contributes to post-transcriptional reprogramming in cancer.

Collectively, these findings highlight a bidirectional regulatory axis in which RNA modifications regulate miRNA processing and function, while miRNAs in turn modulate the expression of RNA-modification regulators to shape oncogenic gene-expression programs.

### Other non-coding ribonucleic acid (snRNA,snoRNA,7SK,caRNA)

Spliceosomal small nuclear RNAs (snRNAs), small nuleolar RNAs (snoRNAs), and the transcriptional regulator 7SK small nuclear RNA undergo extensive epitranscriptomic modifications that influence RNA–protein complex assembly and splicing fidelity. Spliceosomal snRNAs (except U6) carry a 2,2,7-trimethylguanosine cap, whereas U6 possesses a γ-monomethyl guanosine cap, and their internal residues frequently undergo pseudouridylation, 2′-O-methylation, or base methylation such as m^6^A and m^2^G. Pre-mRNA splicing defects are increasingly recognized as a hallmark of cancer. One contributing mechanism involves dysregulation of the cap-modifying enzymes CMTR1 and CMTR2, which generate the Cap1 and Cap2 structures; CMTR2 somatic mutations—present in 5%–6% of endometrial, lung, and melanoma tumors—are associated with widespread splicing alterations [100]. Among snRNAs, U2 is the central catalytic RNA that initiates pre-mRNA splicing, and its interaction with the branch-point adenosine triggers the first transesterification reaction. U2 carries numerous modifications, including pseudouridines (Ψ), 2′-O-methylations (Nm), and a conserved m^6^A residue, most of which are deposited by H/ACA and C/D box snoRNAs. Dysregulated snoRNA programs—such as deletion of KRAS-interacting SNORD50A/B [101] or overexpression or downregulation of SNORD52, SNORD42A, SNORA42, and SNORA23—shift modification states at defined rRNA residues, enhancing translation of metabolic, proliferative, or stemness-associated mRNAs [102]. The 7SK snRNA controls transcriptional elongation by trapping P-TEFb, and m^6^A modifications on 7SK regulate the release of P-TEFb through interactions with m^6^A-reader proteins. Dysregulation of m^6^A writers (METTL3/METTL14) or erasers (ALKBH5/FTO) alters 7SK methylation, promoting aberrant liberation of P-TEFb and hyperactivation of oncogenic transcriptional programs, particularly in MYC-high cancers [103]. Given that CDK9 inhibitors are already in clinical development, integrating 7SK–m^6^A–P-TEFb dynamics may refine patient stratification and therapeutic sensitivity. Malignancy is also characterized by profound dysregulation of chromatin-associated RNAs (caRNAs), including lincRNAs, promoter-associated RNAs, enhancer RNAs, and repeat-derived transcripts, whose functions are dynamically regulated by the epitranscriptome. Specific modifications on these caRNAs act as functional switches that promote oncogenesis. For example, METTL3-dependent m^6^A is deposited on caRNAs, and loss of METTL3 or the nuclear m^6^A reader YTHDC1 stabilizes these transcripts—particularly LINE1-derived RNAs—leading to increased chromatin accessibility and elevated transcription [104]. Furthermore, m^5^C modification of retrotransposon-derived caRNAs regulates chromatin architecture through recognition by the methyl-CpG-binding protein MBD6, which recruits deubiquitinase activity to remove H2AK119ub and promote chromatin opening. TET2 normally erases m^5^C by oxidizing it, and loss-of-function TET2 mutations therefore cause aberrant accumulation of m^5^C-marked caRNAs. This, in turn, drives MBD6-dependent chromatin decompaction and gene activation, creating a dependency of TET2-mutant leukemia on the m^5^C–MBD6 axis. Consistently, MBD6 depletion selectively suppresses the proliferation of TET2-mutant leukemic cells and ameliorates their associated hematopoietic abnormalities [105].

### Non-coding ribonucleic acids and immune evasion

RNA modifications also contribute to immune evasion in cancer. A-to-I editing acts on dsRNA regions, unwinding local secondary structures by replacing adenosines with inosines—a reaction catalyzed by the ADAR enzymes ADAR1 and ADAR2. Among these, ADAR1 is uniquely induced by interferon signaling, and its elevated activity suppresses innate immune surveillance by destabilizing endogenous dsRNA structures and masking them from recognition. In particular, ADAR1-mediated editing relaxes long dsRNA duplexes that would otherwise activate MDA5, thereby allowing cancer cells to evade innate immune activation [106]. Recent findings further show that the m^**6**^A reader YTHDF1 enhances ADAR1 expression by directly binding m^**6**^A-modified ADAR1 transcripts—particularly the IFN-inducible p150 isoform—and promoting their translation. By stabilizing ribosome association with these mRNAs, YTHDF1 increases ADAR1 protein synthesis in response to interferon. Consequently, YTHDF1 knockdown diminishes IFN-induced A-to-I editing, restores dsRNA sensing, and upregulates interferon-stimulated genes. [107]. Collectively, these observations indicate that multiple RNA modifications—through coordinated control of dsRNA structure, ADAR1 expression, and innate immune signaling—operate in a multilayered manner to promote immune escape in cancer, underscoring their potential as therapeutic targets.

### Crosstalk across non-coding ribonucleic acids

Across the transcriptome, RNA modifications are distributed among diverse ncRNA species, forming an intricate and interdependent regulatory landscape. miRNAs can fine-tune the expression of m^6^A writers and readers; snoRNAs both guide Ψ and 2′-O-methylation on rRNA and undergo modification themselves; and tRNA modifications such as mcm^5^s^2^U have been proposed to modulate m^6^A deposition on mRNA. These multilayered interactions reveal a highly integrated modification network that likely co-evolved to coordinate RNA metabolism. Although fully deciphering its collective functional logic remains a major challenge, ongoing studies are gradually uncovering how these interconnected pathways operate together to shape gene-expression regulation.

## Clinical translation

### Diagnostic and prognostic biomarkers

RNA modifications are increasingly measurable in readily accessible specimens and thus attractive for clinical deployment. Ribosomal and transfer RNAs are highly abundant and chemically stable, enabling robust detection of 2′-O-methylation, pseudouridylation, m^5^C, and m^7^G patterns in blood-derived materials. Cancer-associated rRNA ‘signatures’ linked to snoRNA activity or METTL5-dependent m^6^A sites have been correlated with tumor aggressiveness and therapeutic response in multiple entities [108, 109]. tRNA wobble modification profiles (e.g. mcm^5^s^2^U, m^7^G) mirror codon usage biases of oncogenic programs and may stratify patients by translational dependencies [110]. In mRNA, site-specific m^6^A (and its readers) can inform prognosis, immune phenotype, and sensitivity to targeted agents or immunotherapy [111]. From an implementation perspective, assays that aggregate modification signals across abundant RNA classes (rRNA/tRNA) or predefined transcript panels are more reproducible than single-site calls and are amenable to longitudinal monitoring.

### Current therapeutic targets

**Figure 2:**
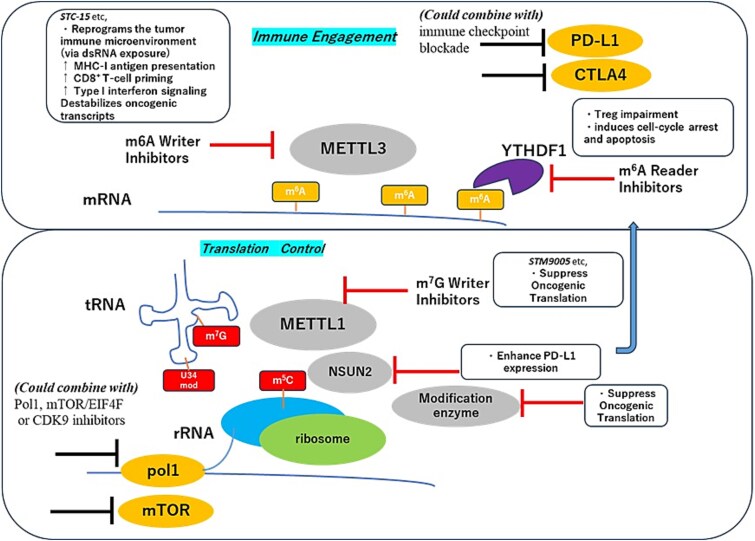
Druggable nodes in the epitranscriptomic machinery enable two complementary therapeutic axes. (Top) METTL3 inhibition reshapes tumor immune visibility by reducing m6A on immune-regulatory transcripts, increasing dsRNA sensing and type I interferon responses, enhancing MHC-I antigen presentation, and improving responsiveness to immune checkpoint blockade. (Bottom) METTL1 and rRNA/tRNA-modifying enzymes support oncogenic translation through ribosome biogenesis and codon-biased elongation; their inhibition collapses the malignant translatome and imposes translational stress, providing a mechanistically orthogonal axis to complement immune engagement.

The therapeutic landscape of RNA modification-guided approaches, encompassing key molecular targets, their mechanistic roles in oncogenesis, and rational combination strategies, is summarized in Fig. 2 and Table 4. Multiple nodes in the epitranscriptomic machinery are druggable.

**Table 4:** Druggable epitranscriptomic targets, mechanisms, agents, and combination strategies in cancer.

Target	RNA modification/class	Mechanism of action (MoA)	Agents	Stage	Combination strategy
METTL3–METTL14	m^6^A (mRNA)	Inhibition reduces m^6^A on oncogenic transcripts, induces differentiation & apoptosis, and impairs leukemia stem-cell maintenance; activates dsRNA–IFN signaling	STM2457; STM3006; STC-15	Preclinical / Phase I	mTOR/EIF4F inhibition; CDK9 inhibition; immune checkpoint blockade (ICB)
METTL1–WDR4	m^7^G (tRNA)	Disrupts codon-biased oncogenic translation leading to tumor fitness loss	STM9005	Preclinical	mTOR suppression; NRF2/KEAP1 pathway inhibition
YTHDF2 (reader)	m^6^A reader (mRNA)	Enhances antitumor immunity via destabilization of regulatory transcripts in Treg	Small-molecule inhibitors	Preclinical	ICB; STING agonists
FBL	2′-O-Me (rRNA Nm)	Alters decoding selectivity and translational output	—	Research	RNA Pol I inhibition; mTOR inhibition
METTL5	m^6^A (rRNA)	Shapes selective translation of growth-program transcripts	—	Research	mTOR/EIF4F inhibition; KRAS pathway inhibition
NSUN2	m^5^C (mRNA/tRNA)	Promotes PD-L1 expression and immune evasion	—	Research	Anti-PD-1/PD-L1; DNA damage agents
U34 pathway (Elongator–CTU–ALKBH8)	tRNA wobble (U34)	Enables codon-biased oncogenic translation under metabolic stress	—	Research	mTOR inhibition; amino acid transporter inhibition; NRF2 inhibition

Among writer enzymes, the m6A methyltransferase METTL3–METTL14 complex represents the most advanced example of therapeutic targeting to date. A first-in-class, highly selective catalytic METTL3 inhibitor, STM2457, demonstrated robust anti-leukemic activity in AML by selectively reducing m6A levels on leukemogenic transcripts, inducing differentiation and apoptosis, and impairing leukemia stem-cell maintenance in vivo) [112]. These studies provided compelling proof of concept that direct inhibition of RNA-modifying enzymes can yield therapeutic benefit in cancer.

Building on this foundation, next-generation METTL3 inhibitors, including STM3006, have been developed through structure-guided optimization, achieving improved potency, selectivity, and pharmacokinetic properties. Preclinical studies summarized in recent medicinal chemistry reviews indicate that METTL3 inhibition by these compounds not only suppresses tumor-intrinsic oncogenic programs but also induces endogenous dsRNA accumulation and activates RIG-I/MDA5–dependent type I interferon signaling, thereby reshaping the tumor immune microenvironment and enhancing antitumor immunity [113].

Importantly, these mechanistic insights have now begun to translate into early clinical evidence. STC-15, a first-in-class oral METTL3 inhibitor developed by Storm Therapeutics, has entered first-in-human clinical evaluation in patients with advanced malignancies (NCT05584111) [114]. In a multi-center, open-label dose-escalation study, STC-15 was generally well tolerated across pharmacologically active dose ranges, with manageable hematologic, dermatologic, and gastrointestinal toxicities. Pharmacodynamic analyses demonstrated rapid and substantial target engagement, with an average ~ 60% reduction of m6A levels on peripheral blood mRNA within 24 hours of dosing. Consistent with preclinical predictions, whole-blood transcriptomic profiling revealed robust activation of innate immune pathways, including type I/II interferon responses and antiviral gene programs. Notably, preliminary signals of clinical activity were observed, including durable partial responses in angiosarcoma and immune checkpoint inhibitor–refractory non–small-cell lung cancer, supporting the concept that METTL3 inhibition can reprogram the tumor immune microenvironment in patients. In addition to STM2457, STM3006, and STC-15, more than seven distinct METTL3 inhibitory chemotypes are currently under preclinical development, underscoring the rapid expansion and sustained momentum of METTL3-targeted drug discovery.

While METTL3 inhibitors have rapidly advanced into clinical development, pharmacological targeting of the tRNA m^7^G writer METTL1 remains at an earlier stage. Nevertheless, accumulating genetic and functional evidence strongly supports METTL1–WDR4 as a next-generation epitranscriptomic target, and structure-guided drug discovery efforts are actively underway. Consistent with this emerging paradigm, the recently described small-molecule inhibitor STM9005 [115] provides the first compelling demonstration that direct pharmacological inhibition of METTL1 is feasible, bioavailable, and therapeutically effective in vivo. STM9005 selectively suppresses m^7^G-modified tRNA populations, disrupts oncogenic translation programs, and exerts potent antitumor activity across multiple aggressive malignancies, including AML, melanoma, and pancreatic ductal adenocarcinoma, with minimal toxicity in preclinical models. Together, these findings establish METTL1 as a bona fide druggable node within the epitranscriptomic machinery and position tRNA m^7^G modification as an attractive target for next-generation cancer therapies.

Reader proteins (e.g. YTH family) modulate mRNA fate and stress adaptation, offering opportunities to rewire oncogenic translation without global transcriptional blockade [116]. Recent work further highlights the therapeutic potential of targeting the m^6^A reader YTHDF2, whose inhibition selectively destabilizes m^6^A-modified transcripts that maintain the survival and suppressive function of intratumoral regulatory T cells, thereby enhancing antitumor immunity without disrupting peripheral immune homeostasis [117]. YTHDF2 inhibitors have been shown to induce cell-cycle arrest and apoptosis, thereby suppressing cancer-cell proliferation [118]. Enzymes governing rRNA modification (FBL [119], METTL5 [120], NSUN family) reshape ribosome selectivity; their inhibition could normalize the translatome or sensitize tumors to existing agents. While efforts are underway to target rRNA biogenesis using RNA Polymerase I inhibitors (e.g. CX-5461) in cancers characterized by hyperactivated translational pathways (e.g. mTOR signaling), this approach carries concerns regarding broad cytotoxicity due to global nucleolar stress. Consequently, targeting rRNA modification enzymes and their regulatory guides, snoRNAs, is gaining traction as a strategy expected to offer superior therapeutic selectivity. Among NSUN family members, NSUN2-mediated m^5^C methylation—facilitated via the NSUN2/ALYREF axis—has been shown to enhance PD-L1 expression and promote immune evasion in non-small-cell lung cancer, underscoring its therapeutic relevance [121]. On the tRNA axis, targeting U34 modification (Elongator–CTU pathway, ALKBH8) may collapse codon-biased translation essential for tumor survival, particularly under mTOR pathway suppression [90]. Selective toxicity is achievable by exploiting tumor-specific dependencies, while normal tissues—with lower proliferative demand—exhibit greater tolerance to partial translational dampening.

### Combination strategies

Epitranscriptomic interventions are likely to be most effective in combination. Many solid tumors show hyperactivated mTOR–EIF4F signaling, which enhances cap-dependent initiation and ribosome biogenesis, while simultaneously upregulating tRNA and rRNA modification pathways (e.g. METTL1–WDR4, NSUN5, FBL, DKC1) to support elongation, decoding fidelity, and selective translation of oncogenic transcripts. In parallel, rRNA biogenesis can also be constrained upstream using RNA Polymerase I (Pol I) inhibitors such as CX-5461, which reduce nucleolar output and sensitize tumors with high translational demand. Therefore, dual-axis blockade of translational initiation and elongation/decoding—for example combining mTOR/EIF4F or CDK9 inhibitors with rRNA/tRNA-modification inhibitors—can convergently suppress oncogenic protein synthesis and collapse codon-biased translation programs. A second complementary axis involves immune engagement, whereby m6A–YTHDF1-dependent antigen cross-presentation in dendritic cells [122], as well as ADAR1-dependent dsRNA editing and innate immune sensing [123], modulate tumor immune visibility and responsiveness to immune-checkpoint blockade. Together, these features enable rational combinations between epitranscriptomic modulators and immunotherapies to enhance antigen presentation, cytotoxic infiltration, and immune checkpoint blockade (ICB) efficacy [121]. Trial designs should incorporate pharmacodynamic markers (e.g. rRNA Nm maps, m^6^A-reader target sets, codon-usage-matched polysome shifts) and early on-treatment liquid-biopsy readouts to confirm target engagement. Collectively, these strategies position RNA-modification–guided approaches as credible components of precision oncology.

## Technologies for detecting ribonucleic acid modifications

Beyond methodological advances, recent innovations in RNA modification detection technologies have fundamentally reshaped how RNA modifications are studied in cancer biology. These approaches have moved the field from bulk, peak-level associations toward increasingly site-resolved, quantitative, and, in some cases, single-molecule–level analyses. As a result, RNA modifications are no longer viewed merely as static epigenetic marks, but as dynamic and context-dependent regulators that can be linked to specific transcripts, cell states, and therapeutic vulnerabilities. In cancer research, these technologies have enabled the discovery of aberrant modification landscapes across mRNAs, rRNAs, tRNAs, and non-coding RNAs, revealed tumor-specific translational programs, and provided mechanistic insight into how dysregulated RNA modifications contribute to oncogenesis, immune evasion, and therapy resistance. Below, we summarize the major technological platforms, emphasizing not only their technical principles and limitations, but also how they have expanded biological and clinical understanding of the cancer epitranscriptome.

Multiple experimental and computational strategies have been developed to detect RNA modifications, including mass spectrometry, antibody- or chemical-based NGS approaches, and nanopore direct RNA sequencing. Each method provides complementary strengths and limitations, as summarized below.

### Mass spectrometry

Mass spectrometry (MS) enables highly quantitative analysis of RNA modifications at the single-nucleotide level [124] and is particularly well suited for distinguishing chemically similar modified nucleosides. High-resolution MS can accurately infer the chemical composition of modifications; however, positional information is lost once RNA is hydrolyzed into nucleosides. To overcome this limitation, tandem MS (MS/MS)–based approaches coupled with high-performance liquid chromatography (HPLC) have been developed to analyze partially digested or intact RNA fragments. In these strategies, RNA is fragmented using specific endonucleases—such as RNase T1, RNase A, or RNase U2—or, in the case of short RNAs such as miRNAs, directly subjected to MS/MS without prior hydrolysis [125]. The resulting oligonucleotides are then separated by HPLC, and fragment ions generated by collision-induced dissociation provide sequence information—typically spanning only a few nucleotides from either terminus—which enables localization of modifications within short RNA fragments.

Despite these advantages, MS/MS-based approaches still face key limitations: software tools for automated interpretation remain underdeveloped, and very short fragments often lack sufficient sequence context to unambiguously assign modification sites. Thus, although tandem MS represents a powerful orthogonal method for confidently identifying modification chemistry, it offers only limited resolution for mapping modification positions across longer RNAs. These approaches are particularly well suited for highly structured RNAs such as tRNAs and rRNAs. Consequently, tandem MS has been central to many classical studies that identified and characterized chemical modifications in these RNA species [126, 127].

### Next-Generation Sequencing (NGS)-based approaches

NGS is a powerful approach for transcriptome-wide detection of RNA modifications; however, information on most RNA modifications—except RNA editing—is typically lost during PCR amplification. Therefore, antibody-enrichment or chemical-based strategies are often combined with NGS. Because the major RNA modifications present in mRNAs include m^6^A, pseudouridine, inosine, and m^5^C**,** methodological development has largely focused on these modifications (Summarized in Table 5). Various approaches have been explored, including antibody-based methods [128], antibody–crosslinking strategies [129], and chemical-labeling–coupled approaches. Antibody-based approaches suffer from variable antibody specificity and typically generate broad enrichment peaks centered around the modification site, making it difficult to achieve true single-nucleotide resolution. One of the earliest examples of chemical-labeling–coupled sequencing was the detection of inosine. Because early RNA-seq platforms had high error rates and whole-genome sequencing remained prohibitively expensive, approaches that relied on sequencing both DNA and RNA to distinguish RNA editing events were costly and inaccurate. These limitations prompted the development of RT-stop and RT-misincorporation methods that exploit artificially introduced chemical labeling. In the case of inosine detection, a synthetic chemical label was used to convert inosine into N^1^-cyanoethylinosine (ce^1^I)**,** which blocks reverse transcription and results in truncation of the cDNA [130]. These chemical-based RT signature approaches were subsequently extended to other RNA modifications [131, 132]. However, because reverse transcription must stall at bulky adducts or introduce characteristic misincorporations, these strategies generally struggle to detect multiple adjacent modification sites or to resolve complex modification patterns.

**Table 5:** Sequencing-based RNA modification detection methods.

	Detection strategies			
Modification	Antibody-based	Chemical labeling	Bisulfite/deamination	Other
m^6^A	m6A-seq/MeRIP-Seq(2012) miCLIP-seq	m6A-SAC-seq (2023)	GLORI (2023)	
Ψ		Ψ-Seq/Pseudo-seq/PSI-seq (2014) BACS (2024)	BID-seq PRAISE (2023)	
Inosine		ICE-seq (2010) Silc-seq (2023)		EndoVIPER-seq(2020)
m^5^C	Aza-IP (2013)	m5C-TAC-seq (2024)	BS-seq (2012)	

Bisulfite chemistry has been used to detect m^5^C modifications in RNA. More recently, high-accuracy methods derived from bisulfite/deamination chemistry—such as GLORI [133] and PRISE [134]—developed for other modification detection. These approaches exploit the selective deamination of unmodified cytidines while leaving methylated cytosines intact, thereby enabling precise, single-nucleotide–resolution mapping of modifications with substantially improved accuracy compared to earlier antibody- or RT-stop–based methods. In addition, a variety of strategies have been developed to further improve sensitivity and specificity, including approaches that employ TET dioxygenases to oxidize methylated cytosines [135], as well as methods that combine specialized endonucleases with chemical labeling [136].

### Application for single cell analysis

The application of RNA modification analysis at the single-cell or spatial level holds great promise for elucidating the roles of RNA modifications in various cell types, such as immune cells. In particular, m^6^A-dependent regulation of Treg stability and T-cell exhaustion—both of which are critical in CAR-T therapy and immune-checkpoint blockade—as well as ADAR1-mediated evasion of MDA5-dependent dsRNA sensing and its interplay with YTHDF1, represent highly valuable avenues for profiling immune responses and anticancer immunotherapy.

However, direct application of nanopore direct RNA sequencing to single-cell or spatial transcriptomics remains technically constrained by its limited throughput and sensitivity. Although nanopore sequencing is a true single-molecule technology, current protocols still require large numbers of cells to obtain sufficient input RNA, making single-cell–scale detection impractical at present. As a result, NGS-based and antibody-based approaches remain the primary methods for single-cell and spatial interrogation of RNA modifications.

Among these emerging techniques, *scDART-seq* [137] employs a proxy-marking strategy in which the YTH domain fused to APOBEC1 induces C-to-U deamination adjacent to m^6^A sites, enabling indirect recording of m^6^A positions at the single-cell level. *m6A-isoSC-seq* [138] extends this concept by applying the strategy to nanopore cDNA sequencing, thereby allowing isoform-specific analysis of m^6^A modifications. Antibody-based methods such as *scm6A-seq* [139] have also been developed to capture m^6^A signals from single oocytes or blastomeres.

### Nanopore direct ribonucleic acid sequencing

Nanopore direct RNA sequencing (DRS) enables the direct analysis of native RNA molecules, outputting electrical current signals as RNA passes through the nanopore. In principle, RNA modifications perturb the ionic current relative to the expected canonical signal, allowing detection of post-transcriptional modifications (PTMs) directly from the raw signal. However, these current differences are often extremely subtle, making reliable detection at the level of individual reads a substantial challenge.

**Figure 3:**
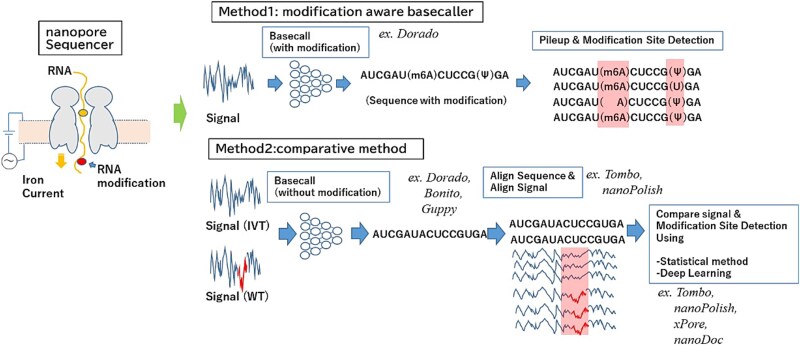
Two major strategies for detecting RNA modifications using nanopore sequencing. Nanopore sequencing enables the detection of RNA modifications through characteristic perturbations in ionic current signals. Two conceptual approaches are commonly used. (1) Basecalling-integrated detection: here, modification signals are incorporated directly into the basecalling model, allowing the potential identification of RNA modifications at the single-read level. Although this strategy offers RNA modification detection on each RNA molecule, it requires sophisticated training on modification-aware models and remains technically challenging. (2) Comparative signal-based detection: in this approach, raw electrical signals from native RNA are compared with signals from unmodified in vitro–transcribed (IVT) RNA to detect modification-induced deviations. This method is broadly applicable and does not depend on the modification type, but it is less suited for read-level assignment and is difficult to accelerate with GPU-based computation.

In nanopore direct RNA sequencing, RNA modifications are detected through characteristic perturbations in ionic current signals as native RNA molecules pass through the pore. Conceptually, two major strategies are used. One approach integrates modification detection directly into the basecalling process, enabling per-read identification of RNA modifications at the single-molecule level. While this strategy provides a powerful framework for resolving modification patterns along individual transcripts, it requires modification-aware training data and complex model optimization. The second approach relies on comparative signal analysis, in which raw electrical signals from native RNAs are statistically compared with signals derived from unmodified in vitro–transcribed (IVT) controls to identify modification-induced deviations. This comparative strategy is broadly applicable and largely agnostic to modification chemistry, but it typically lacks robust read-level assignment and remains computationally demanding. Together, these complementary approaches define the current landscape of nanopore-based RNA modification detection (Fig. 3).

The earliest tools for RNA modification detection, such as *Tombo* [140], realigned raw current signals to the reference sequence and identified modification-associated deviations by comparing wild-type current profiles with those obtained from unmodified IVT or KO controls. Similar approaches have also been implemented in tools such as *Nanopolish* [66], *xPore* [141], and *nanoCompore* [142]. In parallel, methods exploiting base-calling errors induced by RNA modifications were developed, including *ELIGOS* [143], *EpiNano* [144] and related mismatch/indel–based approaches. These tools detect modification sites through statistical comparisons and, by design, have limited ability to distinguish between different types of modifications. Moreover, although they aggregate information across many reads to achieve statistical power, reliably identifying modifications at the level of individual reads—meaning on a single RNA molecule—remains challenging.

As nanopore sequencing technologies improved, more sophisticated deep-learning–based frameworks emerged. For example, *nanoDOC* [145], *nanoDoc2* [146], *m6Anet* [147], *m6ATM* [148] and other machine learning–driven models incorporated convolutional neural networks (CNNs) or recurrent neural networks (RNNs) to identify modification-induced signal perturbations. However, because RNA contains numerous distinct types of modifications, building comprehensive deep-learning models and generating complete training datasets remain challenging. Synthetic training data generated by chemically synthesized modified nucleotides can help, but such synthesis is available only for limited PTMs and cannot capture all sequence contexts with combinations of modified and unmodified bases.

Although tRNAs and rRNAs are relatively short and exist in limited variety, they contain a wide spectrum of chemical modifications. For these RNA species, it is feasible and practical to prepare unmodified IVT RNAs—either through reverse transcription or chemical synthesis—and detect modifications by comparing native RNAs with their unmodified counterparts, making IVT-based computational tools highly useful.

In contrast, mRNAs harbor only a limited set of major modification types, yet must be analyzed across a vast number of transcript species. Generating IVT controls that comprehensively cover this diversity is technically difficult, as transcript abundances vary widely and reverse-transcription reactions often abort prematurely, making it challenging to obtain full-length cRNAs across all targets. For this reason, basecaller-based approaches—most notably Dorado, described in the next section—have emerged as a valuable alternative for mRNA modification detection.

### RNA004 chemistry and dorado basecallers

In 2023, ONT released the RNA004 pore, offering improved accuracy and throughput together with the Dorado basecaller (https://github.com/nanoporetech/dorado), which incorporates modification calling for m^6^A, m^5^C, pseudouridine, inosine, and ribose methylation. Dorado is open source, although the underlying algorithms remain largely unpublished. According to ONT developers, Dorado employs an independent LSTM-based model trained on ~20-nt synthetic oligonucleotides containing defined modifications, enabling classification of modification states. ONT reports per-base accuracies of 97%–99% for these modifications; however, these values are derived from synthetic RNAs and may not reflect the complexity of biological samples. Notably, whereas earlier tools primarily aimed to identify modification sites, Dorado can report multiple modifications at the single-read level, enabling the analysis of modification patterns in relation to isoform choice or splicing. This represents a conceptual shift toward single-molecule, multi-modification profiling.

**Figure 4:**
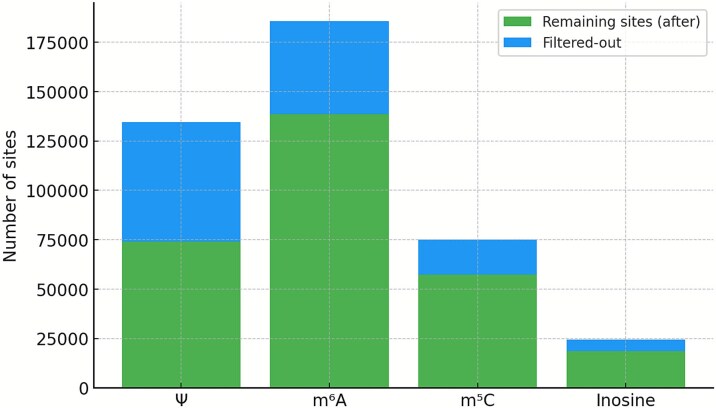
Stacked bar plots show the total number of putative RNA-modification sites reported for each modification type (Ψ, m^6^A, m^5^C, and inosine) and the proportion removed by IVT-based filtering (blue). The remaining sites after filtering are shown in green. As reported by Esfahani et al., a substantial fraction of sites is eliminated when comparing native reads to unmodified IVT controls, indicating that many initial calls reflect noise or sequence-context artefacts rather than true modifications. Notably, the relative abundance of predicted sites across modification types does not match known biological prevalence at the correct order of magnitude, suggesting that a large number of false positives likely remains even after IVT-based filtering.

Esfahani *et al.* [149] reported that Dorado’s pseudouridine model achieved 96%–98% accuracy and F1-scores, while the N^6^-methyladenosine model showed 94%–98% accuracy and 96%–99% F1-scores. Although these values appear robust at first glance, it is important to note that transcriptome-wide application of Dorado yields a very large number of false-positive calls. Esfahani *et al.* demonstrated an approach using unmodified IVT reads to filter out false positives. The fact that substantial numbers of modification calls arise even in unmodified IVT samples itself suggests a high underlying false-positive rate. After filtering with IVT controls, the proportion of sites removed was reported as 45% for Ψ, 25.3% for m^6^A, 23.7% for m^5^C, and 23.4% for inosine (Fig. 4). Given that long, full-length IVT transcripts are difficult to generate and that many regions of endogenous RNAs are not covered by the IVT set, the true false-positive rate is likely even higher. Moreover, the total numbers of modification calls reported—especially for Ψ and m^5^C—are more than two orders of magnitude greater than previously observed with short-read–based methods. Evidence suggests that the detection sensitivity for A-to-I RNA editing in direct RNA sequencing is notably low; therefore, a combined approach using cDNA sequencing may be necessary.

Collectively, these observations indicate that Dorado provides a powerful framework for detecting multiple RNA modifications at the single-molecule level, but its practical use currently requires extensive mitigation of false positives. This includes preparing appropriate unmodified IVT controls, integrating external resources such as RMBase [150] or known motif information, and applying composite filtering strategies to improve specificity. Until highly accurate and sensitive nanopore-based approaches are fully established, it will remain necessary to combine Dorado with conventional methods—including NGS-based chemical or antibody-enrichment approaches and mass spectrometry—to ensure robust and reliable identification of RNA modifications.

## Conclusion

RNA modifications have emerged as a fundamental layer of gene regulation in cancer, extending far beyond their original characterization as static structural marks. Across mRNAs, rRNAs, tRNAs, and diverse non-coding RNAs, dysregulated ‘writer’, ‘eraser’, and ‘reader’ activities reprogram RNA metabolism to promote tumor initiation, metastatic dissemination, therapy resistance, and immune evasion. rRNA and tRNA modifications generate onco-ribosomes and codon-biased translation programs, while context-specific marks on mRNAs and regulatory ncRNAs—such as miRNAs, snRNAs, 7SK, and chromatin-associated RNAs—reshape transcriptional and translational networks in a highly integrated and co-evolved manner.

These insights are beginning to show promise for clinical translation. Modification patterns in abundant RNA species (rRNA, tRNA) and selected mRNA/ncRNA targets are being explored as diagnostic and prognostic biomarkers, and multiple components of the epitranscriptomic machinery—METTL3, METTL1–WDR4, YTH-family readers, rRNA- and tRNA-modifying enzymes, as well as ADAR1—have emerged as druggable nodes. Epitranscriptomic interventions are particularly attractive in combination regimens that converge on translational control or relieve immune suppression, positioning RNA-modification–guided strategies as credible elements of precision oncology.

On the technological front, advances in mass spectrometry, NGS-based chemical and antibody-enrichment methods, and nanopore direct RNA sequencing together provide a rapidly expanding toolkit for mapping RNA modifications. Nanopore-based approaches, especially with RNA004 chemistry and modification-aware basecallers such as Dorado, are pushing the field toward single-molecule, multi-modification profiling. At the same time, high false-positive rates and incomplete training data underscore the need to integrate orthogonal methods and prior knowledge, including IVT controls, known motifs, and curated modification databases. In other words, although comprehensive and fully accurate transcriptome-wide detection of RNA modifications remains an open challenge, the combination of biological insight, clinical translation, and technological innovation is steadily turning the epitranscriptome into a tractable dimension of cancer biology—and a promising source of future biomarkers and therapeutic targets.
